# Automated shape-based clustering of 3D immunoglobulin protein structures in chronic lymphocytic leukemia

**DOI:** 10.1186/s12859-018-2381-1

**Published:** 2018-11-20

**Authors:** Eleftheria Polychronidou, Ilias Kalamaras, Andreas Agathangelidis, Lesley-Ann Sutton, Xiao-Jie Yan, Vasilis Bikos, Anna Vardi, Konstantinos Mochament, Nicholas Chiorazzi, Chrysoula Belessi, Richard Rosenquist, Paolo Ghia, Kostas Stamatopoulos, Panayiotis Vlamos, Anna Chailyan, Nanna Overby, Paolo Marcatili, Anastasia Hatzidimitriou, Dimitrios Tzovaras

**Affiliations:** 1 Information Technologies Institute, Centre for Research and Technology Hellas, 6th km Harilaou-Thermi Road, Thessaloniki, Greece; 2 Institute of Applied Biosciences, Centre for Research and Technology Hellas, 6th km Harilaou-Thermi Road, Thessaloniki, Greece; 3 Carlsberg Research Laboratory, Copenhagen, Denmark; 4 Center for Biological Sequence Analysis, Technical University of Denmark, Copenhagen, Denmark; 5 Department of Immunology, Technical University of Denmark,Genetics and Pathology, Science for Life Laboratory, Uppsala University, Uppsala, Sweden; 6 Karches Center for Chronic Lymphocytic Leukemia Research, The Feinstein Institute for Medical Research, Manhasset, NY USA; 7 Masaryk University, Central European Institute of Technology, Brno, Czech Republic; 8 Hematology Department and HCT Unit, G. Papanikolaou Hospital, Thessaloniki, Greece; 9 Nikea General Hospital, Hematology Department, Piraeus, Greece; 10 Department of Informatics,Ionian University, Corfu, Greece; 11 IRCCS San Raffaele Scientific Institute and Università, VitaSalute, San Raffaele, Division of Experimental Oncology, Milan, Italy

**Keywords:** CLL protein clustering, 3D protein descriptors, descriptor fusion

## Abstract

**Background:**

Although the etiology of chronic lymphocytic leukemia (CLL), the most common type of adult leukemia, is still unclear, strong evidence implicates antigen involvement in disease ontogeny and evolution. Primary and 3D structure analysis has been utilised in order to discover indications of antigenic pressure. The latter has been mostly based on the 3D models of the clonotypic B cell receptor immunoglobulin (BcR IG) amino acid sequences. Therefore, their accuracy is directly dependent on the quality of the model construction algorithms and the specific methods used to compare the ensuing models. Thus far, reliable and robust methods that can group the IG 3D models based on their structural characteristics are missing.

**Results:**

Here we propose a novel method for clustering a set of proteins based on their 3D structure focusing on 3D structures of BcR IG from a large series of patients with CLL. The method combines techniques from the areas of bioinformatics, 3D object recognition and machine learning. The clustering procedure is based on the extraction of 3D descriptors, encoding various properties of the local and global geometrical structure of the proteins. The descriptors are extracted from aligned pairs of proteins. A combination of individual 3D descriptors is also used as an additional method. The comparison of the automatically generated clusters to manual annotation by experts shows an increased accuracy when using the 3D descriptors compared to plain bioinformatics-based comparison. The accuracy is increased even more when using the combination of 3D descriptors.

**Conclusions:**

The experimental results verify that the use of 3D descriptors commonly used for 3D object recognition can be effectively applied to distinguishing structural differences of proteins. The proposed approach can be applied to provide hints for the existence of structural groups in a large set of unannotated BcR IG protein files in both CLL and, by logical extension, other contexts where it is relevant to characterize BcR IG structural similarity. The method does not present any limitations in application and can be extended to other types of proteins.

## Background

The concept of molecular similarity underlies a methodology where molecules are grouped together based on their biological effects, physicochemical properties and three-dimensional structures [1]. Considering that the three-dimensional (3D) protein structure plays a pivotal role in protein functional characterization [2], the comparison of the three-dimensional (3D) molecular structures is a key technique in a variety of applications such as protein function prediction, computer aided molecular design, rational drug design and protein docking [3].

In the absence of known structure, alternative approaches such as comparative modeling can provide a 3D model of a protein, related to at least one experimentally determined protein structure. The most comprehensive examples of these approaches are SCOP [4] and CATH [5], protein structure classification databases that were established to address the evolutionary relationships between protein structures. They are widely used as a benchmark for novel protein structure comparison methods and as a training dataset for machine learning algorithms focused on protein structure classification and prediction [6]. Their rationale is that protein structures are conserved during evolution and the existence of a protein family would facilitate the identification of related proteins through similarities in their structures [7].

Techniques that define similarity between 3D structures can be classified into three categories, i.e. (1) superposition of protein structures where alignment between equivalent residues in not given a priori [8], (2) feature representation of protein spatial profile in multidimensional vectors [9] and (3) time series formed by the alteration of the protein tertiary structure [10].

In the first category, the structural similarity is determined by scaling, rotation, transformation and then super-positioning [11]. Numerous scoring functions have been proposed towards the definition of the positional deviations of equivalent atoms upon rigid-body superimposition. Aligners were implemented with the ability to identify similarities between proteins with large conformational changes. Various metrics for comparing and scoring identity between two protein structures are employed but the most commonly used are p-values [12] and root mean square deviation (RMSD) [2]. Highlighted aligners in this category are represented in Table 1. Although this type of approach is very effective, it is a computationally expensive and time consuming method.

**Table 1:** Distance metrics that measure the average distance between the atoms of superimposed proteins

Similarity metric	Method or software
RMSD	MAMMOTH [55], LGA/GDT [56]
*p*-value	[57]
SAS score & GSAS score	[58]
TM-score	TM-align, Fr-TM-align [59]
S score	MatAlign [60]
STRUCTAL score	LOVOalign [61]
Q-score	SSM [62]

The second approach includes all the shape-based methods. In shape-based approaches, the protein is treated as a 3D object and represented by a multidimensional vector that uniquely characterizes the object. Consequently the comparison between feature vectors is characterized by lower complexity and higher accuracy. Similarity search is committed through global or local features. The global features are computed by the transformation of euclidean space into a metric space that measures the pairwise distances between the points of the 3D objects. The global features are invariant to the deformations of the 3D object. The local features are computed on each key-point of the surface by accumulating pairwise relations among oriented surface points into a local histogram [3].

The last method is related to the comparison between time series. According to this type of methodology, protein structures are translated into polygonal chains [13]. The aforementioned transformation of the 3D object to a feature vector reduces object complexity and it can be handled as a time series [10]. Protein tertiary structures, such as the alpha-carbon atoms along the backbone of a protein, essentially form a 3D polygonal chain and a natural measure for comparing the geometric similarity estimates the similarity between the structures.

The current work aims to categorize chronic lymphocytic leukemia (CLL) patients based on their 3-dimensional protein structures of the clonotypic B cell receptor immunoglobulin (BcR IG) amino acid (IG) sequences following on the shape-based approach. In the paragraphs that follow, the corresponding state-of-the-art analysis is presented.

Geometrical descriptor vectors able to achieve very fast comparisons especially for applications of virtual screening are described in [14, 15]. Spin Images [16] and Shape Histograms [17] are methodologies in molecular surface representation [18]. The former is related to local 2D descriptors while the later exploits the global geometric properties of the molecules. Efforts for the implementation of multi-view methods [19, 20] of molecular surface representation were proved insufficient in proteins in cases without symmetries. Computational approaches that address the significance of small variations between 3D protein structure of high similarity are conducted in the methodologies of [21–23].

Pattern recognition establishes approaches that extract moments from the 3D object. Zernike moments were applied in [24] for a feature representation on a Position-Specific Scoring Matrix (PSSM). Zernike moments descriptors were utilized to extract features in each protein PSSM forming a 42-dimensional feature vector. Finally, machine learning methods called PCVM where applied to accomplish classification. A similar method that implements Legendre moments to predict protein-protein interactions is described in [25]. Zernike descriptors provide a rotation invariant ability to the protein shape comparison as they do not necessitate structural alignment. Additionally, they allow other characteristics of a protein surface, such as electrostatic potentials, to be incorporated into the descriptor vector [26]. Zernike moments can be applied in several problems related to protein structures with satisfactory results. Regarding moment extraction, 2D Polar-Fourier coefficients [27] and 2D Krawtchouk moments [28] create a rotation invariant feature vector by taking as input the volume of the 3D object. Spherical Harmonics [29] are widely used for a large scale of structural similarity comparison. Although the formation of an orthonormal 1D vector allows fast comparisons, they present an inaccuracy in the results that is connected to the alignment parameters.

Rigid object methodologies are inherently limited by ignoring the flexibility of the molecule. To overcome this limitation, approaches that respect the shape deformation of molecules were utilized. The two main categories on feature extraction of non-rigid approaches are: a) global-shape-based [30, 31] and b) local-shape-based methods [32–34]. The former usually is used to create a metric where Euclidean space or Euclidean metrics are transformed into pairwise distances between points of the 3D object surface. The aforementioned points are invariant to deformations of the 3D object. The final descriptor vector is formed by the feature histograms of the distances. Local-shape-based methods sample the surface and extract descriptors for each of the sampled local regions. When the local descriptors are extracted then a feature-based methodology is implemented in order to translate them into global. Besides the discriminative ability between the proper local shape descriptors, they also satisfy significant criteria such as fast descriptor extraction, compactness and rotation invariance.

The motivation of this study was to examine the abilities of global and local 3D descriptors for pairwise distance calculation, instead of applying bioinformatics-specific similarity scores. We hypothesized that their high capability in describing general 3D structures could be applied to the comparison of 3D protein structures. The structures for analysis emerged from the primary sequences of the clonotypic BcR IG of patients with CLL.

CLL is the most common adult leukemia, with still unclear etiology. That said, primary and 3D structure-based reasoning strongly implicates antigen selection in disease ontogeny and evolution [35, 36]. Molecular categorization of CLL patients based on BcR IG sequence similarity has so far been addressed using bioinformatics methods of structural similarity calculation [35, 37]. The novelty of the approach proposed herein lies in the combination of current state-of-the-art bioinformatics methods with the extraction of features arising from 3D object recognition methods. The most up-to-date 3D prediction structure algorithms were implemented to construct patients’ models. The proposed combined methodology achieves an efficient grouping of CLL patients in accordance to their biological and clinical features, especially in light of the recently identified stereotyped subsetsb [37, 38]. The results confirm the original hypothesis that the combination of bioinformatics-specific techniques, such as TM-align, and general-purpose 3D descriptors achieves a high discriminative power compared to using only bioinformatics-specific methods.

## Methods

**Fig. 1:**
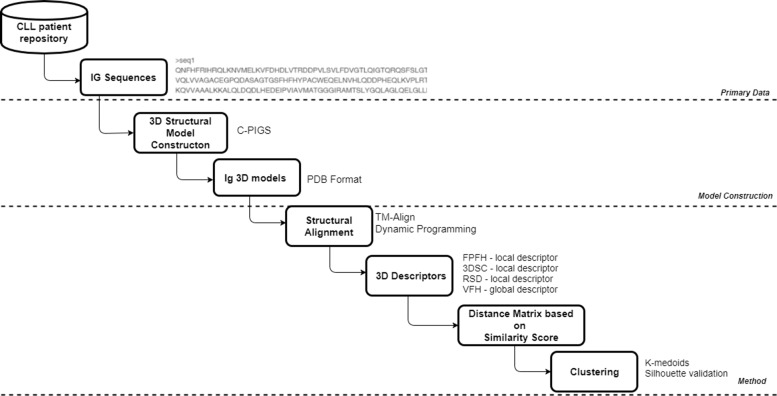
Block diagram illustrating the proposed methodology

In Fig. 1, a pipeline diagram presents the layers that compose the proposed methodology. The method is separated in three main levels, i.e (1) primary data collection, (2) creation of the 3D protein structures and (3) 3D protein structure comparison method. A baseline method raised from pure bioinformatics approaches was utilized as a benchmark for validation.

Extensively, the baseline benchmark consists of the following steps. First, the original BcR IG heavy and light amino acid sequences of 925 CLL patients were transformed to 3D protein models, using state-of-the-art protein structure prediction tools. This resulted in a set of protein models in PDB format. For each pair of models, structural alignment was carried out and a structural similarity score was computed. The TM-align and TM-score algorithms were used for the alignment and the similarity score computation, respectively. A similarity matrix was formed to consider the similarity scores between every pair of models. The matrix was was ultimately used to organize the proteins into clusters, using various existing clustering methods.

The proposed method modified this baseline approach by replacing the bioinformatics related TM-score to a similarity metric between a pair of models with 3D descriptors originated from the field of 3D object recognition. Specifically, the Fast Point Feature Histograms (FPFH) [39], 3D Shape Context (3DSC) [40], Radius-based Surface Descriptors (RSD) [41], Viewpoint Feature Histograms (VFH) [42] and a combination of the above local descriptors where applied to the 3D structures in order to extract the appropriate features for the comparison. The descriptors were compared using the root mean square deviation (RMSD) distance metric. The distance for every pair of models was computed and all results were incorporated into a distance matrix, which was finally used to model clustering.

### Structural alignment

The TM-align algorithm [43] identifies the best structural alignment between protein pairs by combining the TM-score rotation matrix and Dynamic Programming (DP). When comparing two protein structures, the second model is rotated and translated appropriately, until the maximum alignment between the two structures is achieved [44]. The Kabsch algorithm, a method for calculating the optimal rotation matrix that minimizes the RMSD between two paired sets of points performed the structure comparison.

The alignment process includes alignment over the secondary structures of the BcR IG sequences, based on the gapless matching of the IG sequences, and alignment using an equally weighted combination of the previously extracted results. Heuristic iterations were applied, meaning that the steps were repeated until the alignment became stable and the highest TM-score was achieved. The TM-score is calculated as:

1 $$ {TM}_{score} = Max{\left [ \frac{1}{L_{Target}} \sum\limits_{i=1}^{L_{align}}{\frac{1}{1+\left(\frac{d_{i}}{d_{0}(L_{Target})}{}\right)^{2}}} \right]}  $$

where, *L*_*Target*_ is the length of the target protein that the source protein is aligned to, *L*_*align*_ is the number of aligned parts, *d*_*i*_ is the distance between the *i*_*th*_ pair of residues, and *d*_0_ (*L*_*Target*_) is given by the equation:

2 $$ d_{0}({L_{Target}})=1.24^{3}\sqrt{L_{Target}-15}-1.8  $$

### 3D descriptors used

The proposed method is based on the extraction of 3D descriptors from the raw PDB files. Considering a pair of PDB files, they are first aligned in space and then 3D descriptors are extracted from them. The descriptors capture specific geometric properties of the distribution of the atom positions. The following four types of descriptors have been considered:

- Fast Point Feature Histogram (FPFH - local descriptor)
- 3D Shape Context (3DSC - local descriptor)
- Radius-based Surface Descriptor (RSD - local descriptor)
- Viewpoint Feature Histogram (VFH - global descriptor)

The above descriptors are briefly described in the following sections. In order to apply the descriptors, the proteins are considered as 3D point clouds, where each point of the cloud corresponds to a protein atom. For the conducted experiments presented in this paper, the implementations contained in the PCL C++ library [45] were used for these descriptors.

#### Fast point feature histogram (FPFH)

The Fast Point Feature Histogram (FPFH) [39] represents the angular variations in the neighborhood of each point in the input point set. It is a modification of the Point Feature Histogram (PFH) descriptor [46], towards faster computation and smaller size. The PFH descriptor considers the *k*-neighborhood around each query point i.e. atom. For each pair of points in the neighborhood, a coordinate system is formed by the pair of points and the surface normals at these points, and 3 measures of angular variation between the points and the normals are computed. The histogram of these measures computed for all pairs of points in the neighborhood constitutes the PFH descriptor for the query point.

In the FPFH descriptor, instead of considering all pairs of points within the neighborhood of the query point, only the relationships between the query point and its neighbors are initially considered. The resulting histograms constitute an intermediate Simplified Point Feature Histogram (SPFH) descriptor for each point. The final FPFH descriptor is computed by adding the SPFH descriptors of the neighbors of the query point, weighted by their inverse distance to the query point:

3 $$ \text{FPFH}(p) = \text{SPFH}(p) + \frac{1}{k} \sum_{i=1}^{k} \frac{1}{d_{i}} \text{SPFH}(p_{i}),  $$

where *p* is the query point, *k* is the number of its nearest neighbors, *p*_*i*_ is the *i*-th nearest neighbor point, and *d*_*i*_ is the distance between *p* and *p*_*i*_.

The resulting FPFH descriptor for a query point is a 33-dimensional vector, being more compact than the original PFH descriptor, as well as faster to extract. The FPFH descriptor is invariant to position, scale and orientation, as the original PFH descriptor. However, due to numerical limitations, FPFH may not be completely invariant to rotation in certain cases. For this reason, the protein files are aligned prior to computing the FPFH descriptors, in order to ensure that there is no rotational variance.

#### 3D shape context (3DSC)

The 3D Shape Context (3DSC) [40] descriptor describes the distribution of points around each point of a point cloud. Considering each point *p* of the point cloud, the support region of *p* is defined as a sphere centered at *p*. The orientation of the sphere is determined by the surface normal at *p*. The support region is divided into bins, determined by uniform divisions in the two angular dimensions (azimuth and elevation) and logarithmic divisions along the radial dimension.

The 3D division of the support region defines the bins of a histogram of point counts within this region. The value of the (*i*,*j*,*k*) bin, where *i*, *j* and *k* are indexes of the angular and radial position of the bin, is computed by counting the points of the point cloud that fall within the bin. However, the contribution of each point *p*_*i*_ within the support region is weighted by the following factor:

4 $$ w(p_{i}) = \frac{1}{\rho_{i} \sqrt[3]{V(i, j, k)}},  $$

where *V*(*i*,*j*,*k*) is the volume of bin (*i*,*j*,*k*) and *ρ*_*i*_ is the density of the points around *p*_*i*_. The density is computed by counting the points within a sphere of radius *δ* around *p*_*i*_.

#### Radius-based surface descriptor (RSD)

The Radius-based Surface Descriptor (RSD) [41] describes the region around a point by approximating the local surfaces with spheres and estimating the minimum and maximum radii of the fitted spheres. Considering a point *p* and a point of its neighborhood *p*_*i*_, the two points can be thought as lying on a sphere of radius *r*. Since infinite spheres pass through the two points, the one that also respects the surface normals at the two points is selected. If the distance between the two points is *d* and the angle between their surface normals is *α*, then the radius of the fitted sphere can be computed using the formula for determining the length of a sphere chord:

5 $$ d = r \sqrt{2 - 2\cos{a}}  $$

For computational efficiency reasons, the radius is computed from *d* and *α* using just the first terms of the Taylor expansion of the above equation. After computing the radii of the spheres for every neighbor of point *p*, the minimum and maximum ones are kept and used as the descriptor for *p*, thus obtaining a very compact yet discriminating descriptor.

#### Viewpoint feature histogram (VFH)

The three above descriptors are local descriptors, i.e. they compute a vector representation for each point in the point cloud of the 3D object considered. The Viewpoint Feature Histogram (VFH) [42] is a global descriptor, describing the whole point cloud. The VFH descriptor is conceptually based on the FPFH descriptor. Instead of computing FPFH descriptors for each point of the point cloud and its neighborhood, a single FPFH descriptor is computed for the object’s centroid, considering all the points of the point cloud as its neighbors.

This central descriptor constitutes one part of the VFH descriptor. The other part considers the histogram of the angles between the normals at each point of the point cloud and a fixed direction, determined by a fixed viewpoint, outside the point cloud. First, the vector from the viewpoint to the object’s centroid is computed and then the angles between this vector and each of the normals of the point cloud are used to construct an angle histogram. This histogram constitutes the second part of the VFH descriptor. Considering a fixed direction from the viewpoint to the centroid, instead of considering the direction from the viewpoint to each point in the point cloud ensures scale invariance. However, the VFH descriptor is not rotation invariant, since it depends on the object’s pose relative to the viewpoint. However, the advantage of the VFH descriptor is the compactness offered by a global descriptor, as it represents the whole object with a single vector.

### Distance matrix calculation

After extracting the 3D descriptors from a pair of aligned proteins, a distance measure can be calculated between them, quantifying their structural differences in the corresponding descriptor space. In the general case, a descriptor extracted from protein *i*, is a set of vectors *F*_*i*_={**f**_*i*,1_,**f**_*i*,2_,…,**f**_*i*,*L*_}, where *L* is the number of points in the protein’s 3D model. The feature vector for point *k* of protein *i* is a vector $\mathbf {f}_{i,k} \in \mathbb {R}^{D}$, where *D* is the descriptor dimensionality, which is generally different for different descriptor types. The above formulation fits well with the local 3D descriptors, such as FPFH, 3DSC and RSD, since they consist of a feature vector for each point in the 3D point cloud. However, the same formulation can be used for global descriptors, such as VFH, as well, if the global descriptor is considered as a local descriptor extracted from only a single point. Thus, in the following, the same formulation is used for all types of descriptors.

In order to compare between the descriptors of two proteins, a distance measure that can assess the difference in space between two sets of points needs to be used. The Root Mean Square Deviation (RMSD) distance metric has been used hereby for this purpose. The RMSD metric is commonly used for the comparison between protein structures, by computing an average of the point-to-point differences among the protein atoms. However, hereby it is not used to compare the actual 3D coordinates of the atoms, but instead the high-dimensional coordinates of the extracted feature vectors for each pair of points. The RMSD distance metric between proteins *i* and *j* is defined as follows:

6 $$ \text{RMSD}(F_{i}, F_{j}) = \sqrt{\frac{1}{L} \sum\limits_{k=1}^{L} ||\mathbf{f}_{i,k} - \mathbf{f}_{j, k}||^{2}},  $$

where ||·|| denotes the Euclidean distance. In case that the descriptor type is a global one, such as VFH, i.e. *L*=1, the RMSD metric is reduced to the Euclidean distance between the descriptors vectors. The smaller the RMSD metric, the closer the corresponding proteins are, in terms of their similarity with respect to the corresponding descriptor type.

The RMSD distance is computed between every pair of proteins in the considered dataset, so that a square symmetric distance matrix is computed. This distance matrix can then be provided as input to clustering algorithms.

### Clustering methods

In order to cluster the proteins in groups of similar characteristics with respect to the various types of descriptors considered, the following clustering methods have been used:

- *k*-medoids
- hierarchical agglomerative
- DBScan

The **k-medoids** method is similar to the *k*-means clustering method, with the difference that it uses a distance matrix as input instead of vectorial representations of the objects to cluster. The *k*-means method proceeds by guessing at the cluster center positions within the feature space, and gradually updating them, in order to fit better with the available data. In *k*-medoids, no feature space is defined, so there is no notion of cluster center positions. Instead, only a distance matrix is provided as input, encoding the similarities and differences among objects. In such a case, the role of cluster centers is played by representative objects from the set of objects themselves, called *medoids*. At the beginning, the set of medoids is selected arbitrarily, e.g. randomly, from the set of objects in the dataset. The objects are grouped by assigning each object to the closest one, in terms of the distance matrix used. Then, through an iterative procedure, the medoids are updated, selecting more representative objects, so that they are better fitted with the other objects. Considering each group of objects, the object with the smallest sum of distances from the other objects in the group is selected as the group medoid.

The **hierarchical agglomerative** clustering method is a bottom-up clustering approach, building gradually larger clusters of data, in a hierarchical manner. At the beginning, each data point is considered as a separate cluster. At each iteration, the two clusters that are nearest to each other are merged into a single cluster. The procedure continues until all clusters are merged into a single cluster, containing all data points. The distance between two clusters is hereby defined as the mean value of the pairwise distances between each pair of points in the two clusters. Hierarchical clustering results in a tree-like representation of the data. Cutting the tree at a specified height results in clustering the data at different granularities. In this paper, the height of the cutting is determined by specifying the number of desired clusters.

**DBScan** is a density-based clustering method, which does not require the number of clusters to be known from the beginning. It starts from an arbitrarily chosen point and considers its *ε* neighborhood. If it is in a dense part, it forms a cluster, also cotaining the *ε* neighborhoods of its neighbor points. The procedure is repeated for each point in the cluster, until no other point can be considered as being near the points of the cluster. Then, another point is selected, to begin a new cluster. DBScan uses two parameters: the neighborhood size *ε* and the minimum number of points in the neighborhood, in order to characterize the neighborhood as dense.

All the above clustering method do not require the objects to be represented by vectors; they only need distances to be defined between objects. This fits well with the local 3D descriptors used hereby, since a protein is not represented by a single feature vector, but rather by a set of feature vectors, one for each point in the protein model. However, the representation as sets of vectors does allow the definition of distances among proteins, e.g. using the RMSD measure, as described above, which makes the above methods suitable. Another approach would be to use the distance matrix as the input to methods such as multidimensional scaling, in order to map the proteins to points in a low-dimensional space, before performing traditional clustering methods such as *k*-means. However, this could potentially lead to information loss, if the selected space dimensionality does not correspond to the underlying intrinsic dimensionality of the points. Using the distance matrix directly as input overcomes this issue.

The *k*-medoids and the hierarchical agglomerative methods require the number of clusters to be a priori provided as an input parameter. However, in the exploratory task of examining a set of proteins for clusters, the number of clusters to be discovered is not known. A method that can also determine the number of clusters in the data is needed. In this paper, this issue is overcome by performing the clustering several times, considering a range of number of clusters and selecting the one that maximizes a certain clustering quality criterion. The average silhouette width has been used hereby as this clustering quality criterion. Considering a protein *i*, let *α*_*i*_ be the average distance of protein *i* to all other proteins of the same cluster. Let also *b*_*i*_ be the minimum of the average distance of protein *i* to the proteins of all other clusters. The silhouette width for protein *i* is defined as:

7 $$ s_{i} = \frac{b_{i} - \alpha_{i}}{\max{\{\alpha_{i}, b_{i}\}}}  $$

The silhouette width takes values in the range from -1 to 1. Values close to 1 mean that *b*_*i*_ is large and *α*_*i*_ is small, which means that object *i* is very close to the other objects of its cluster, while at the same time it is far away from the objects of the other clusters. This in turn means that object *i* has been correctly clustered. On the other hand, values close to -1 mean that the object would be more properly assigned to another cluster. Considering the average silhouette width for all objects provides a measure of the clustering quality. Large values of the average silhouette width mean that the clustering produced compact and clearly divisible clusters. Thus, seeking for the number of clusters that achieves the largest average silhouette width is equivalent to seeking for the number of clusters that is most appropriate to describe the underlying data.

### Combination of descriptors based on their clustering performance

In addition to the individual descriptor types (FPFH, 3DSC, RSD and VFH), a combination of them has also been considered. The various descriptor types are diverse in form, since they may be local or global and also contain vectors of different dimensionalities. This makes the process of combining them not straightforward. However, since the input to the clustering algorithm is not the descriptors themselves but rather the distance matrices produced from them, a natural way to combine the multiple descriptors is by merging their corresponding distance matrices.

Let **D**_*m*_ be the distance matrix associated with descriptor type *m*. The combined distance matrix **D** is defined as the weighted sum:

8 $$ \mathbf{D} = \sum\limits_{m} w_{m} \mathbf{D_{m}},  $$

where the sum is over all descriptor types considered. Two different approaches have been examined for the definition of the weights used in the sum. The first approach is to consider them all equal to 1. This creates a distance matrix where the distance for a pair of proteins is the sum of the distances computed for this pair using the various descriptor types. Taking this sum implicitly considers that all descriptor types are equal in terms of discriminating power. In reality, some descriptor types may be more suitable for clustering the protein datasets than others. In order to handle such differences, the second approach is to consider unequal weights for the sum, ones that reflect the discriminating power of the descriptors. Hereby, the average silhouette widths of the clusterings produced by the different types of descriptors have been used as the weights. The higher the average silhouette width of a clustering, the more fitting the clustering is for the underlying data, i.e. the more descriptive the corresponding descriptor may be for the protein set. Thus, using a high average silhouette width as a weight for the sum, would mean that more importance is given to the corresponding descriptor while computing the combined distance matrix.

Note that the average silhouette width of a clustering also depends on the number of clusters. The maximum average silhouette width computed for a descriptor type, using a range of cluster numbers has been used as the weight for this descriptor.

## Results

For the experimental evaluation of the proposed methodology, two different protein model datasets were formed. The clusters were evaluated externally and internally in order to obtain cluster accuracy and quality respectively.

### Datasets

The datasets derive from BcR IG sequences obtained from 925 CLL cases diagnosed according to the iwCLL criteria [47]. Following established bioinformatics methods, 137/925 cases were found to belong to subsets with stereotyped i.e. (quasi)-identical BcR IG, hereafter designated as stereotyped subsets [48, 49]. As a first step, we examined a dataset including BcR IG sequences from cases belonging to six well-characterized stereotyped subsets. As a second step, we analyzed BcR IG sequences from all cases, stereotyped and non-stereotyped. The first dataset was deployed as ground truth for the evaluation of the proposed method. Hence the second dataset formed for unsupervised clustering applications. The number of CLL sequences in each dataset is described in Table 2. The subset size distribution of the dataset consisting of only the stereotyped BcR IG is summarized in Table 3.

**Table 2:** Datasets description

Dataset	Patients	Predefined subsets
D1	137	6 (D1.A ∼ D1.F)
D2	925	N/A

**Table 3:** Subset size distribution in the annotated dataset

Subset	Type	Size
1	IGHV clan I/IGKV1(D)-39	38
2	IGHV321/IGLV3-21	42
4	IGHV4-34/IGKV2-30	22
6	IGHV1-69/IGKV3-20	12
7	IGHV1-69/IGLV3-9	12
8	IGHV4-39/IGKV1(D)-39	11

Regarding the BcR IG structure prediction, the C-PIGS method was used, which is based on the Prediction of Immunoglobulin Structure (PIGS) [50] approach. In the PIGS web server, antibody VL and VH framework regions were used as input parameters. The sequence identity within both chains was examined with the threshold of 80%. If the aforementioned criterion was not satisfied then the two templates with the highest sequence similarity, measured by the Blosum24 score [51] of both the light and heavy chain were utilized. The H3 loop was always modeled using the template with the best sequence similarity; the other loops were modeled using a different template only if the corresponding loop in the framework template did not display the same length and canonical structure of the target loop. Finally, the C-PIGS models were built by remodeling the H3 loop of the PIGS models using the template identified by the approach developed in [52]. An out-standing study of the customized PIGS methodology is described by [53].

### Clustering of annotated proteins

In order to evaluate the accuracy of clustering using the 3D descriptors, a first round of experiments was conducted, using the annotated dataset. Each type of 3D descriptor was used to cluster the data into 6 clusters, as many as the ground truth subsets in the annotated dataset. The resulting clusterings were compared to the ground truth clustering, i.e. the one where each cluster corresponds to the established protein subset. The same procedure was also performed using the TM-score for clustering.

The *k*-medoids, Agglomerative Hierarchical clustering and Density-based spatial clustering of applications with noise (DBSCAN) methods were used to cluster the data using the extracted descriptors. After extracting descriptors from each pair of aligned proteins, the RMSD distances between each pair of descriptors is computed, forming a square distance matrix. This matrix is used as the input to the clustering methods.

In order to compare the resulting clusterings to the ground truth clustering, the Rand index was used. The Rand index measures the number of agreements between the two compared clustering, over all pairs of points. Considering a set of *N* objects *S*={*o*_1_,*o*_2_,…,*o*_*N*_}, e.g. a set of proteins, and two partitionings of this set, *X*={*X*_1_,*X*_2_,…,*X*_*m*_}, partitioning the objects into *m* groups, and *Y*={*Y*_1_,*Y*_2_,…,*Y*_*n*_}, partitioning the objects into *n* groups, the Rand index is computed as follows:

9 $$ R = \frac{a + b}{{{n}\choose{2}}},  $$

where *a* is the number of pairs in *S* that are grouped in the same cluster in *X* and in the same cluster in *Y*, while *b* is the number of pairs in *S* that are grouped in different clusters in *X* and in different clusters in *Y*. The denominator is the number of pairs of objects in *S*, and is equal to $\frac {n(n-1)}{2}$. In other words, the Rand index measures the percentage of pairs that have been clustered in the same way in both clusterings, over all possible pairs of objects. The Rand index takes values from 0 to 1, with 1 meaning that the two clusterings are the same, while 0 means that the two clusterings are completely different.

The results of the multiple clusterings are summarized in Table 4. With the exception of the VFH descriptor, all individual 3D descriptors manage to produce clusterings that are very close to the ground truth one, achieving accuracies from 77 to 89.5%. The accuracies of the 3D descriptors are larger than the accuracy achieved with the method using the TM-score for clustering. This shows that exploiting the 3D structural information encoded in 3D descriptors commonly used in the area of object recognition achieves an improvement compared to using traditional structural information. Regarding the individual 3D descriptors, the RSD descriptor achieves the highest accuracy. The VFH descriptor achieves the least accuracy compared to the other descriptors. This can be attributed to the fact that it is a global type of descriptor, hence a lot of information regarding local variations of the points in the protein is discarded, thus losing discriminating capacity.

**Table 4:** Comparison of clustering accuracy (Rand index) between TM-score and the various 3D descriptors (6 clusters) for the 137 protein structures

**Method**	K-medoids	Agglomerative	DBScan
TM-score	85.40%	58.25%	71.23%
FPFH	89.10%	86.59%	88.40%
3DSC	88.00%	78.60%	86.20%
RSD	89.5%	77.32%	84.67%
VFH	83.20%	65.62%	76.31%
Combined Silhouette Weights	**89.70**%	**87.42**%	88.67%
Combined Equal Weights	89.00%	85.51%	**88.82**%

The highest accuracy is highlighted

The table also includes the results produced using the combined distance matrix, considering either equal weights for the combination or weights based on the silhouette width. The combination of descriptors achieves the maximum accuracy for all types of clustering considered, reaching 89.7% compared to the ground truth for the *k*-medoids clustering. This demonstrates that combining 3D descriptors manages to produce clusterings that are more accurate than using the individual descriptors. This is an expected result, since inaccuracies of one clustering can be filtered out when considering multiple clusterings at once.

The comparative analysis of the clustering methods also demonstrated that the agglomerative and DBScan methods achieved lower accuracy than the *k*-medoids method. For this reason, the *k*-medoids method is selected for the rest of the present study.

**Fig. 2:**
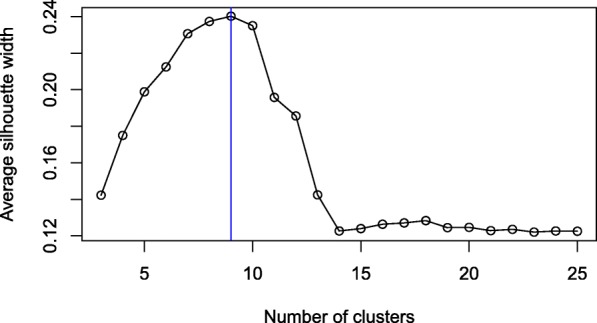
Determination of optimal number of clusters for the FPFH descriptor

In the results of Table 4, a fixed number of 6 clusters was considered for clustering using any of the descriptor types; it was chosen as this is the number of stereotyped subsets existing in the annotated dataset. However, some subsets may be further subdivided into smaller categories, due to finer differences between their BcR IGs, which were not reflected in the annotation by experts. In order to compensate for this, the same experiments were repeated, but using an optimal number of clusters for each type of descriptor. This optimal number of clusters was computed based on an internal measure of cluster compactness, namely the average silhouette width, as described in the “Methods” section. Considering a single type of descriptor, several clusterings were computed, using a range of number of clusters from 3 up to 25, in order to determine the number of clusters that achieves the largest average silhouette width. An example of such a determination of the optimal number of clusters is depicted in Fig. 2, for the FPFH descriptor, where the number of clusters achieving the largest average silhouette width is 9.

Table 5 summarizes the clustering accuracy results for the annotated dataset, when using the optimal number of clusters, computed separately for each descriptor type. Allowing the number of clusters to vary provides more freedom to the clustering algorithm to cluster the data based on their intrinsic clusters. This allows smaller clusters to emerge, resulting in larger numbers of clusters than the ground truth ones, ranging from 7 to 9. However, this freedom allows the clustering algorithms to group the proteins more accurately, thus resulting in higher values for the Rand index. Using this type of analysis with the silhouette-based combination of the descriptors achieves a 92.2% accuracy with respect to the ground truth protein separation.

**Table 5:** Comparison of clustering accuracy between TM-score and the various 3D descriptors (optimal number of clusters) for the 137 protein structures

Method	Num. clusters	Rand index
TM-score	8	89.7%
FPFH	9	89.3%
3DSC	9	89.5%
RSD	7	92.0%
VFH	8	85.3%
Combined silhouette weights	7	**92.2**%
Combined equal weights	7	90.2%

The highest accuracy is highlighted

**Fig. 3:**
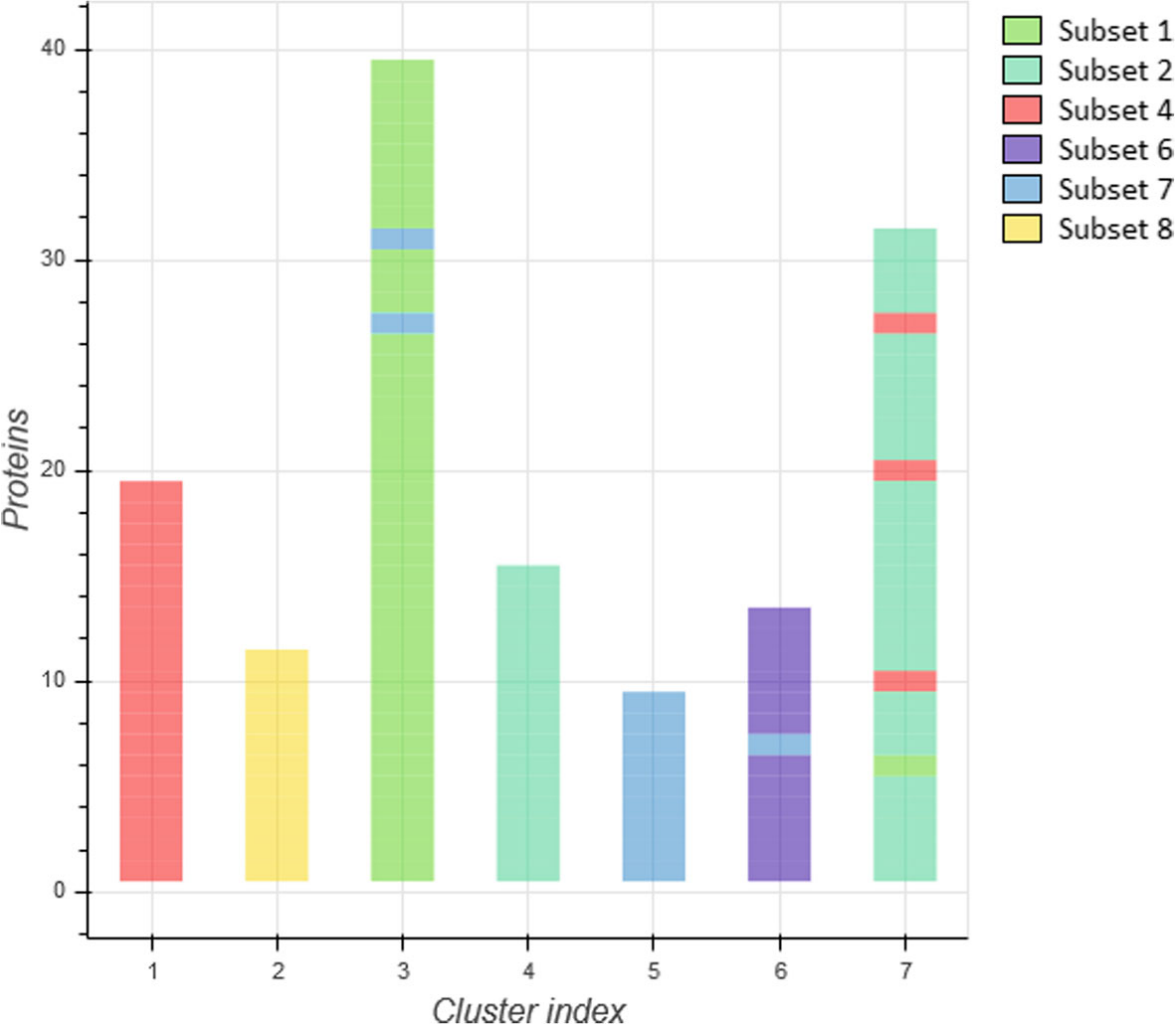
Clustering of the annotated protein dataset, using the combined descriptors method

As an illustration of the clustering performance, Fig. 3 presents the computed clusters graphically. Each vertical bar corresponds to one of the computed clusters. Each bar is constructed from small rectangles, each one representing a single protein. The higher a vertical bar, the more proteins are contained in the corresponding cluster. The colors assigned to each protein correspond to the different ground truth subsets. It can be observed that, most of the proteins have been correctly clustered, with few exceptions. Moreover, the clustering method discovered 7 clusters, instead of 6, splitting stereotyped subset #2 (green-blue color) into two clusters (indexes 4 and 7). The reason behind this separation is probably the pattern of somatic mutations in the immunoglobulin heavy-chain variable region gene (IGHV).

### Clustering of all BcR IGs

The procedure followed for clustering the annotated dataset was repeated, this time using the whole BcR IG protein dataset, including both stereotyped (annotated) and non-stereotyped (non annotated) cases. For each type of descriptor, the optimal number of clusters was first determined, using the maximum average silhouette width method. Then, the proteins were clustered using the *k*-medoids method with the optimal number of clusters.

The performance of the various clusterings was evaluated using two types of measures. The first is the average silhouette width itself, which is a measure of the cluster compactness and separation. In general, clustering is based on the assumption that the underlying data form compact clusters of similar characteristics. Larger average silhouette width means that the result of a clustering algorithm consists of compact clusters which are well separated from each other, i.e. probably close to the actual data distribution. A small average silhouette width means e.g. that one of the clusters discovered by the clustering algorithm could be separated in two clusters, or that some of the discovered clusters could be merged together. The average silhouette width is an internal evaluation measure, in the sense that it uses only information contained in the dataset, without assuming any knowledge of ground truth class labels or clusterings.

The second type of evaluation measure is the Rand index, which is an external measure, in the sense that it makes use of ground truth knowledge. The evaluation using the Rand index is similar to the evaluation of the annotated dataset in the previous section, by comparing the produced clusterings to the ground truth clustering. However, only the annotated BcR IG were used for the computation of the Rand index. In other words, after computing a clustering of all proteins, both annotated and unannotated, we wanted to evaluate how well they have been clustered by examining the clustering distribution of the annotated ones, within the whole clustering. The results of the clustering are summarized in Table 6.

**Table 6:** Comparison of clustering accuracy between TM-score and the various 3D descriptors (optimal number of clusters) for the 925 protein structures

Method	Num. clusters	Avg. silhouette width	Rand index
TM-score	4	0.001	60.0%
FPFH	14	0.070	88.9%
3DSC	13	0.057	89.3%
RSD	9	0.056	83.9%
VFH	7	0.006	76.3%
Combined silhouette weights	15	0.071	90.2%
Combined equal weights	14	0.069	**90.8**%

The highest accuracy is highlighted

To evaluate the quality of the applied clustering methods of BcR IG 3D models, we included in our cohort 137 cases originating from 6 CLL stereotyped subsets namely subsets #1, #2, #4, #6, #7 and #8. The reason for this approach was that (i) stereotyped, highly homologous BcR IG primary sequences are anticipated to produce overall similar 3D structures, hence providing a reference for evaluating the developed workflow; and, (ii) these subsets are well characterized in terms of both biological and clinical properties [38]. Subset size distribution was as described in Table 3.

Next, we assessed the reasons behind the separation of subsets #1 and #2 into 2 different clusters each. As mentioned above, stereotyped subset #1 sequences express different IGHV genes that belong to the same phylogenetic clan. Indeed, the utilization of a different IGHV gene was most likely the reason behind the separation of subset #1 models. In more detail, 7/8 (88%) of subset #1 models assigned to cluster 3 expressed the IGHV5-10-1 gene, whereas 27/29 (93%) of subset #1 models in cluster 6 utilized genes belonging to the IGHV1 gene subgroup. In regard to subset #2 models, the reason behind the separation was probably the pattern of somatic mutations within the IGHV gene. More specifically, 9/15 (66%) of subset #2 models from cluster 4 exhibited the presence of somatic mutations within the FR1 region of the Ig heavy chain sequence. In contrast, none of the subset #2 models in cluster 9 carried such a mutation.

**Fig. 4:**
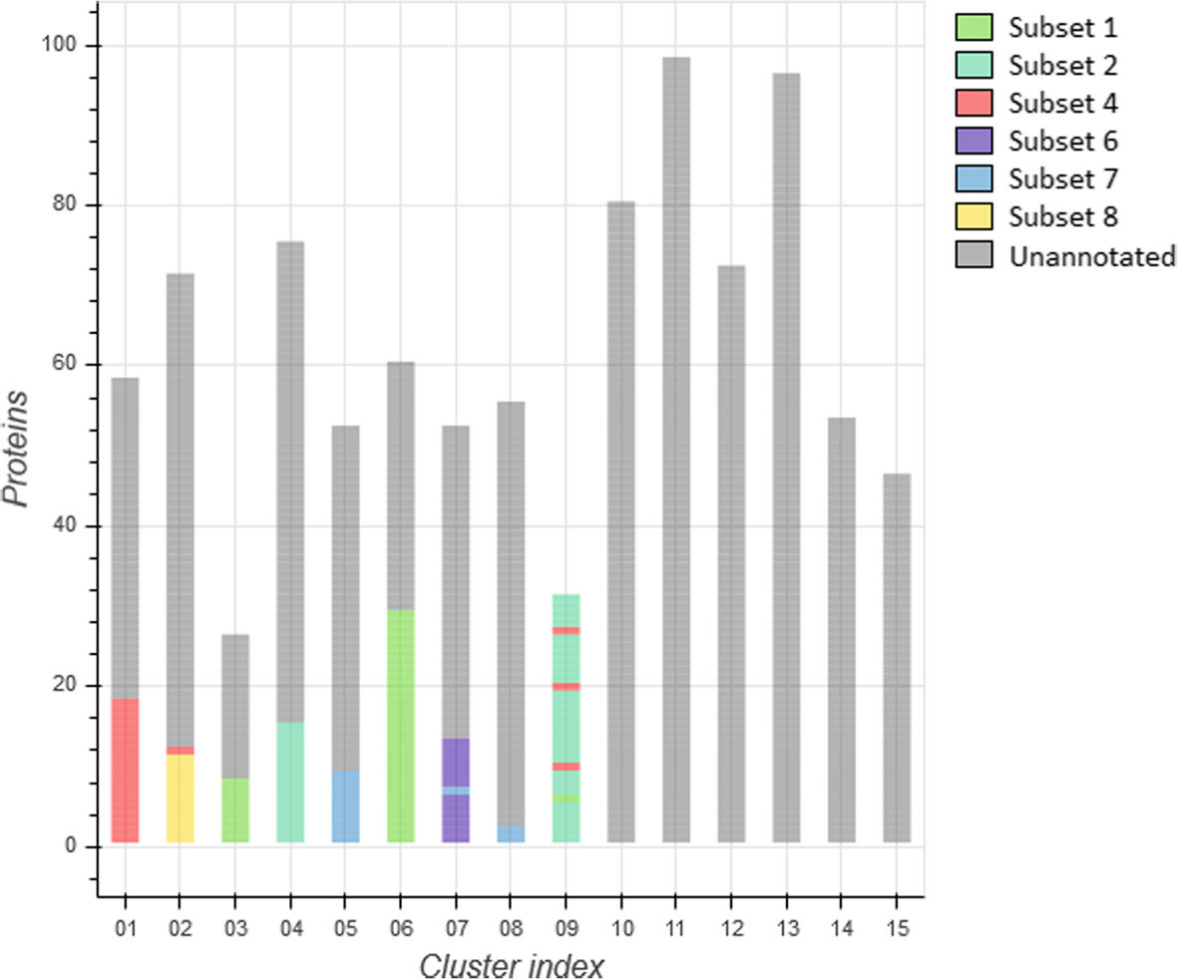
Clustering of both the combined annotated and unannotated protein dataset, using the combined descriptors method
- cluster 2 contained 11/11 (100%) subset #8 models along with a single (4%) subset #4 model,
- cluster 3 contained 8/38 (21%) subset #1 models,
- cluster 4 contained 15/42 (36%) subset #2 models,
- cluster 5 contained 9/12 (75%) subset #7 models,
- cluster 6 contained 29/38 (76%) subset #1 models,
- cluster 7 contained 12/12 (100%) subset #6 models and a single (8%) subset #7 model,
- cluster 8 contained 2/12 (17%) subset #7 models, and finally,
- cluster 9 contained 27/42 (64%) subset #2 models, a single (3%) subset #1 model as well 3/22 (14%) subset #4 models.

We assessed the efficacy of each individual clustering algorithm as well as the “combined” method regarding their potential of biological significance through evaluating the distribution of these 137 stereotyped BcR IG 3D models across different clusters. At the level of individual descriptors, the best results were observed in the case of the 3DSC clustering method, yet, the most robust results were obtained through the combined approach. In detail, the combined method led to the assignment of stereotyped BcR IG 3D models in 9/15 clusters with the following distribution, as also illustrated in Fig. 4:

- cluster 1 contained 18/22 (82%) subset #4 models,

Therefore, we focused our analysis on the results obtained with the combined method. Relevant to mention, our cohort of non-stereotyped IG models was representative of CLL in terms of BcR IG heavy and light gene repertoire properties and, thus, largely informative. In specific, IGHV3 gene subgroup cases predominated (395/788, 50.1%) followed by IGHV4 (186/788, 23.6%) and IGHV1 cases (151/788, 19.2%). In regard to the IG light chain expression, around 2/3 of cases expressed kappa light chains (507/788, 64.3%), as reported for CLL. As mentioned above, the "combined" clustering method ended up with the formation of 15 clusters. The distribution of BcR IG 3D models was not equal among different clusters whose size ranged from 26 to 98 models (average: 52.5, median: 53 models). Interestingly, cluster 9 consisted exclusively of 31 stereotyped models, whereas clusters 10-15 did not contain any stereotyped Ig 3D models.

In detail, cluster 1 besides containing the majority (82%) of subset #4 models included mostly (38/40, 95%) non-stereotyped BcR IG 3D models of the IGHV4 gene subgroup. From these, most models (25/38, 66%) expressed the IGHV4-34 gene, as in the case of subset #4 models. Cluster 2 comprised all subset #8 models and a single (4%) #4 model. This was not unexpected, since both subsets express IGHV4 subgroup genes (IGHV4-39 versus IGHV4-34, respectively) and carry the IgG heavy chain isotype, in itself a rarity for CLL [54]. Besides stereotyped models, cluster 2 contained IGHV4 models (51/59, 86%) from which the majority expressed the IGHV4-39 gene, which is characteristic for subset #8. Clusters 3 and 6 contained subset #1 models as well as non-stereotyped models utilizing IGHV genes from the same phylogenetic clan (Clan I: IGHV gene subgroups 1, 5 and 7) with the frequencies being 100% and 84%, respectively. Clusters 4 and 9 consisted of subset #2 models. Non-stereotyped models assigned to cluster 4 utilized IGHV genes from the IGHV3 subgroup (85%) with the most frequent gene being IGHV3-21 (7/51, 14%) as in subset #2, whereas cluster 9 did not contain any non-stereotyped models. Cluster 5 contained the majority of subset #7 models (75%). Non-stereotyped models in this cluster expressed BcR IG utilizing IGHV1 subgroup genes, predominated by IGHV1-69, the hallmark of subset #7. Cluster 7 contained all subset #6 models and a single model of subset #7: a possible explanation for this is that both subsets express the IGHV1-69 gene. Again, non-stereotyped models mostly expressed the IGHV1-69 gene. Cluster 8 contained a small fraction (17%) of #7 models. In this case, the same trend was not followed and most non-stereotyped models expressed IGHV3 genes with the most prominent being the IGHV3-23 gene. Clusters 10, 11 and 13-15 comprised IGHV3-expressing models yet different genes predominated in each cluster: IGHV3-23, IGHV3-7, IGHV3-30, IGHV3-48 and IGHV3-30, respectively. Finally, cluster 12 contained models of the IGHV4 subgroup with the most frequent gene being IGHV4-34. In terms of light chain usage, we observed a dominance of either the kappa (clusters 1-3, 5-8, 10-12, 14) or the lambda (clusters 4, 13, 15) light chain.

According to our results, the clustering of BcR IG 3D models reflected to very great extent the classification of IG molecules based on the primary sequences of both the heavy and the light chains of the Ig molecule. Indeed, each individual cluster was characterized by the predominance of a single IGHV gene subgroup and a specific light chain isotype.

## Discussion

In this work, a novel method of clustering BcR IG protein 3D structures is introduced. The method underlines the significance of the combination between classic bioinformatics methods with 3D descriptors that goes beyond the realm of bioinformatics. The proposed methodology relies on a combination approach based on local descriptors. Two approaches of combination have been used, one using equal weights and another using silhouette widths as weights. Both performances perform better than using the individual descriptors. However, a clear comparison between the two approaches cannot be made yet. When clustering the 137 annotated protein structures, the silhouette-based approach achieved higher accuracy, while the equal weights approach achieved slightly higher accuracy when clustering the 925 structures. Further investigation on the suitability of each approach for different datasets is considered as future work.

Methodology evaluation succeeded through the dataset separation to ground truth and test set. The ground truth was formed by the well-established CLL stereotyped subsets and the test set from the unlabeled structures that result from C-PIGS methodology. As final analysis level, a clustering of significant external accuracy and internal quality resulted. Overall, our findings support that the innovative workflow described here enables robust clustering of 3D models produced from BcR IG primary sequences from patients with CLL. Furthermore, they indicate that CLL classification based on stereotypy of BcR IG primary sequences is likely also verified at the IG 3D structural level. More generally, this approach can be implemented to the analysis of BcR IG amino acid sequences in any domain of immunology ranging from normal, autoreactive and malignant B cell populations.

## Conclusions

A novel BcR IG protein 3D structure comparison technique is proposed for determining the local structural similarity between the 3D models. The method’s generalizability was demonstrated by applying it to two different datasets: one labeled, formed by 137 protein structures that belong to six well-established CLL stereotyped subsets, and one mainly unlabeled, formed by 925 (including the cases that belong to stereotyped subsets) protein structures.

Local and global 3D descriptors where tested and the optimal combination of the local descriptors was selected, based on their performance regarding the average silhouette width. The combination of the local-based descriptors derived from the structurally aligned parts is used to compute an overall distance matrix, which is then used as input for the clustering procedure. The combined descriptor presented Rand Index 89.7% and 92.2% in clustering the labeled data to six and the optimal number of clusters, respectively. The higher accuracy in the optimal cluster number is justified by the biological meaning in data. Additionally, the clustering results of the unlabeled data revealed 90.8% accuracy in the optimal cluster numbers. These results support that the innovative workflow described here enables robust clustering of 3D models produced from BcR IG sequences from patients with CLL. The established methodology can be expanded in different types of 3D protein structures.

The selection of the appropriate 3D descriptor is an issue worth studying further. In future work, methods of time series analysis in combination to 3D descriptors will be examined. More specifically the implementation of Fréchet distance and Dynamic Time Warping will be examined on estimating the distance between the 3D models in combination to 3D descriptors.

## Abbreviations

3DSC: 3D shape context
CLL: Chronic lymphocytic leukemia
FPFH: Fast point feature histogram
IGHV: Immunoglobulin heavy chain variable region
PIGS: Prediction of immunoglobulin structure
RSD: Radius-based surface descriptor
VFH: Viewpoint Feature Histogram
